# Democratic Backsliding Based on Illusions: Authoritarians' Biased Perception of Media Freedom Contributes to Their Political Support

**DOI:** 10.1002/ijop.70126

**Published:** 2025-10-26

**Authors:** Márton Hadarics

**Affiliations:** ^1^ Institute of Psychology ELTE Eötvös Loránd University Budapest Hungary

**Keywords:** authoritarianism, freedom of speech, motivated perception, multilevel modelling, political support

## Abstract

Authoritarianism plays a pivotal role in shaping anti‐democratic preferences, including support for institutional restrictions on free speech. In this study, we present empirical evidence that authoritarianism undermines public discourse not only through such antidemocratic views but also by fostering ignorance regarding problems with freedom of speech. The study aims to demonstrate that authoritarianism is associated with a more positive perception of media freedom, particularly in contexts where such freedom is more problematic. Using multilevel structural equation modelling and representative data from 31 European countries in the European Social Survey (*N* = 59,685), we found that authoritarianism correlates with perceptions of media freedom (*b* = 0.45; *p* < 0.001), and indirectly, through this perception, with political support—indicated by political trust (*b* = 0.12; *p* < 0.001), satisfaction with democracy (*b* = 0.16; *p* < 0.001) and satisfaction with the government (*b* = 0.13; *p* < 0.001). Moreover, the association between authoritarianism and perceived media freedom is stronger in countries with weaker actual media freedom (*b* = −0.03; *p* = 0.016). These findings underscore how authoritarianism contributes to support for antidemocratic regimes that curtail the boundaries of public discourse, often fueled by biased perceptions of free speech opportunities.

## 1 Introduction

Institutionally, free media serves as the fourth estate, and legally, the civil right to freedom of speech is an essential requirement for a well‐functioning democratic political system. To achieve this well‐functioning system, people's support for free speech is crucial in modern democracies. Consequently, psychological research has taken a keen interest in uncovering both the nature and the psychological foundations of attitudes towards censorship and free speech (Drieghe et al. 2023; Suedfeld et al. 1994; Zakharin and Bates 2024; Zhang et al. 2015). Authoritarianism consistently correlates with attitudes towards free speech (Altemeyer 1996; Costello et al. 2022; Downs and Cowan 2012; Suedfeld et al. 1994). This relationship is unsurprising, given that authoritarianism has long been considered one of the most significant dispositional bases for antidemocratic beliefs and political preferences (Adorno et al. 1950; Duckitt 2001; Feldman and Weber 2023; Fromm 1941; Stenner 2005). Nonetheless, besides establishing more lenient beliefs about centralised censorship and the restriction of free speech, forming positively biased perceptions about the actual functioning of free speech rights is another potential way authoritarianism might damage democracy. This study presents empirical evidence demonstrating that authoritarianism is associated with constraints on open public discourse. This association is partly reflected in a divergence between favourable perceptions of media freedom and a more restrictive, antidemocratic reality. A lack of sensitivity to issues concerning media freedom appears to align with a preference for maintaining existing conditions among individuals with authoritarian orientations. A central objective of the present research is to emphasise the contextual nature of authoritarianism by illustrating that in environments characterised by limited media freedom, authoritarianism is linked to a wider gap between perceived and actual media freedom. This dynamic contributes to the persistence of an illusion of freedom in contexts where it is substantively absent.

### 1.1 Authoritarianism and Free Speech Attitudes

Authoritarianism, as a psychological disposition, has been conceptualised in various ways over the decades. A common thread in notable theories is the profound need of authoritarians for social order, security, and stability—an antithesis to the desire for personal freedom, independence and open‐mindedness (Adorno et al. 1950; Altemeyer 1996; Duckitt 2022; Feldman and Weber 2023; Fromm 1941; Stenner 2005). This psychological trade‐off often results in a simultaneous preference for social and cultural uniformity on one hand and a preference for the status quo (as opposed to social change) on the other. Established social norms and conventions provide the stability, security, and predictability sought by authoritarians. Consequently, they tend to hold positive attitudes towards the established social status quo and its institutions. Simultaneously, they perceive non‐conventional social groups that challenge this cultural and institutional establishment as significant threats (Osborne et al. 2023). Given their emphasis on cultural uniformity and adherence to social conventions, it's unsurprising that authoritarianism is associated with a willingness to restrict the rights and opportunities of groups that deviate from these norms (Feldman et al. 2021). As a result, authoritarianism predicts a range of anti‐democratic and illiberal policy preferences, including disregard for human rights, civil liberties, equality before the law, legal procedural justice and even the negation of the rule of law (Duckitt 2022; Feldman and Weber 2023). Since social uniformity is threatened by dissident and non‐conventional voices that challenge the status quo and advocate for social change, it's no surprise that authoritarians strongly support restricting such voices in public discourse. They have consistently shown approval for legal limitations on free speech and centralised censorship (Altemeyer 1996; Costello et al. 2022; Downs and Cowan 2012; Suedfeld et al. 1994). In contrast, intellectual humility—an antithesis of authoritarian dogmatism—correlates with a commitment to cultivating freedom of speech (Drieghe et al. 2023; Zakharin and Bates 2024).

### 1.2 Motivated System Attitudes in Different Contexts

Authoritarianism, as a psychological disposition, is characterised by a profound need for order and stability. Consequently, authoritarians tend to favour the status quo over rapid social changes. This preference often leads to positive attitudes towards established social, institutional, and economic systems, including political support, institutional trust and system justification (Dunn 2020; Ma and Yang 2014; Pattyn et al. 2012; Pernia 2022; Vargas‐Salfate et al. 2018). However, recent research suggests that the impact of motivational factors driving positive system attitudes can vary based on context. For instance, individuals' need for security, certainty, or belief in a just world tends to enhance positive perceptions of the system in countries where the actual system performance and operation are more problematic (Hadarics and Kende 2025, Hadarics and Krekó 2025). This implies that motivational foundations play a stronger role in negative contexts, creating a wider gap between the problematic reality and the positive perceptions that overlook its flaws (Hadarics 2025).

The tendency of authoritarians to support the status quo may clash with their preference for antidemocratic or illiberal policies, depending on the context. In less inclusive or democratic regimes, authoritarians can simultaneously satisfy their desire for an antidemocratic system and stability (as opposed to change). However, achieving this balance becomes more challenging in well‐functioning, inclusive democracies. Additionally, it's essential to recognise that democracy has become a global normative expectation. Many autocratic and illiberal regimes strive to maintain the illusion of democracy both internally and externally (Bozóki 2013; Roth 2009). By doing so, they downplay the significance of democratic principles, such as freedom of speech, and may even ignore or deny practical issues. For instance, a recent study found that authoritarianism correlated with more positive perceptions of liberal democracy's functioning in more illiberal countries (Hadarics and Krekó 2025). This highlights how authoritarians can simultaneously justify the status quo and their illiberal preferences by misperceiving and disregarding democratic deficits.

### 1.3 A Potential Authoritarian Misperception of Free Speech Opportunities

If authoritarianism serves as a motivational foundation for ignoring democratic flaws, it's reasonable to extend this to the malfunctioning of freedom of speech and media restrictions. Authoritarians may favour political systems that silence dissenting voices, and a psychological strategy to justify such systems could involve motivated ignorance regarding free speech limitations. Simultaneously, authoritarians might be less supportive of systems that allow a wide range of opinions in public discourse, as this diversity could undermine their need for certainty and cultural uniformity. In our study, we aim to gather empirical evidence supporting the idea that authoritarianism is related to a divergence between the actual functioning of freedom of speech and its perceived state. Furthermore, we explore how this biased perception mediates the association between authoritarianism and the political support for regimes that restrict free speech opportunities.

## 2 The Current Study

To test our assumptions, we analysed data from the 10th round of the European Social Survey (ESS 2023), extended with the World Press Freedom Index (WPFI; Reporters Sans Frontières 2024). The ESS is an international survey program that aims to reveal the attitudes, beliefs, and values of the European public regarding various social issues. The 10th round was registered in 31 countries (with the participation of almost 60,000 respondents arranged in probabilistic representative national samples), and it contained multiple items assessing different dimensions of political support, authoritarianism, and the perception of media freedom. The WPFI is a country‐level and ranking measure of media freedom developed by the international organisation of Reporters Sans Frontières to assess the ‘ability of journalists as individuals and collectives to select, produce, and disseminate news in the public interest’ (Reporters Sans Frontières 2024). The participating countries and the most important demographic characteristics of the national samples can be found in Table 1.

**TABLE 1:** Most important demographic characteristics of the national samples.

Country	Gender		Age		Education level (ISCED)		Household income		Religiousness		Left–right ideology	
	Male (Count)	Female (Count)	Mean	SD	Mean	SD	Mean	SD	Mean	SD	Mean	SD
Austria	945	995	49.935	17.742	4.422	1.695	6.140	2.648	4.009	2.869	4.484	2.157
Belgium	672	669	48.987	19.121	4.590	1.888	6.046	2.437	4.233	3.316	5.137	2.067
Bulgaria	1284	1434	52.680	18.256	4.483	1.658	4.943	2.643	4.466	2.799	5.382	2.572
Switzerland	782	741	49.595	18.862	4.185	1.788	5.650	2.673	4.671	3.133	5.041	2.062
Cyprus	376	498	47.098	16.787	5.383	1.637	5.298	2.595	5.302	2.866	5.527	2.606
Czechia	1079	1397	48.299	17.719	4.292	1.459	5.148	2.689	2.199	2.847	5.699	2.128
Germany	4056	4289	50.315	19.103	4.455	1.686	6.067	2.734	3.615	2.963	4.342	2.060
Estonia	693	849	51.647	18.566	4.769	1.637	6.038	2.525	3.407	3.050	5.522	1.963
Spain	1088	1194	49.438	18.063	3.966	2.240	5.245	2.764	3.547	3.065	4.019	2.909
Finland	780	797	52.609	19.323	4.652	1.768	6.048	2.725	4.487	2.933	5.633	2.264
France	974	1003	49.544	18.722	4.076	1.851	6.262	2.987	4.716	3.501	5.071	2.248
United Kingdom	506	643	55.709	18.292	4.227	2.119	5.394	2.944	3.491	3.092	4.973	2.165
Greece	1335	1464	50.380	16.974	4.051	1.707	5.085	1.881	6.294	2.327	5.269	1.962
Croatia	715	877	50.262	18.780	3.927	1.589	5.295	2.761	5.615	3.025	5.197	2.530
Hungary	699	1150	50.489	18.768	3.682	1.386	5.190	2.786	4.140	3.012	5.791	2.415
Ireland	840	930	53.458	18.283	4.220	1.954	4.784	2.733	5.070	3.029	5.252	2.145
Israel	539	664	41.569	17.463	5.055	1.589	4.511	2.869	4.661	3.708	5.123	3.102
Iceland	435	468	50.144	18.774	4.412	1.903	5.307	2.517	4.992	3.054	5.039	2.345
Italy	1254	1386	51.586	18.690	3.582	1.765	4.926	2.401	5.298	2.751	5.134	2.352
Lithuania	638	1021	51.420	18.126	4.556	1.634	4.984	3.017	5.090	2.877	5.332	2.573
Latvia	418	592	49.372	18.394	5.276	1.514	5.548	2.658	3.564	2.767	5.578	2.288
Montenegro	648	630	47.062	17.665	3.983	1.483	4.797	2.401	5.685	2.725	3.637	3.136
North Macedonia	649	780	51.446	17.627	3.638	1.530	4.318	2.415	6.992	2.436	5.448	2.894
Netherlands	750	720	48.625	18.502	4.394	2.027	6.455	2.645	4.065	3.310	5.136	2.108
Norway	720	691	47.311	18.165	4.834	1.724	5.741	2.717	3.205	2.811	5.014	2.428
Poland	998	1067	48.986	18.928	4.136	1.925	5.499	2.885	5.221	3.013	5.558	2.881
Portugal	772	1066	54.051	18.467	3.020	2.153	4.831	2.592	5.530	2.954	4.904	2.114
Serbia	783	657	53.375	17.613	4.403	1.528	6.125	2.892	5.525	3.055	4.561	2.816
Sweden	1099	1181	52.108	19.862	4.416	1.847	6.220	2.571	2.658	2.697	5.126	2.636
Slovenia	591	661	49.411	18.985	4.046	1.485	5.267	2.638	4.752	3.069	4.937	2.416
Slovakia	647	771	53.075	16.759	4.108	1.501	5.250	2.371	6.103	3.193	5.036	2.627

Abbreviations: ISCED, International Standard Classification of Education; SD, standard deviation.

### 2.1 Measures

#### 2.1.1 Authoritarianism

Three ESS items were used to measure individual‐level authoritarianism. Two items were measured with a 1–5 scale (‘Obedience and respect for authority are the most important values children should learn’; ‘What [Country] needs most is loyalty towards its leaders’; 1 = agree strongly; 5 = disagree strongly), while the third on a 0–10 scale (‘How acceptable for you would it be for [Country] to have a strong leader who is above the law?’; 0 = not at all acceptable; 10 = completely acceptable). The scoring of the 5‐point scales was reserved to indicate stronger authoritarianism.

#### 2.1.2 Perceived Media Freedom

Respondents indicated their perceptions of media freedom on a 0–10 scale (‘The media in [Country] are free to criticise the government’; 0 = does not apply at all; 10 = applies completely).

#### 2.1.3 Political Support

Three dimensions of political support were applied: three items for political trust (trust in the national parliament, legal system, and the police), one for satisfaction with the national government, and another for satisfaction with democracy. All these items were measured on a 0–10 scale (trust items: 0 = no trust at all; 10 = complete trust; satisfaction items: 0 = extremely dissatisfied; 10 = extremely satisfied).

#### 2.1.4 National Media Freedom

The actual level of media freedom was quantified by the 2020 WPFI. Based on both factual data and expert ratings, the WPFI indicates media freedom on a 0–100 scale in the case of each country (Reporters sans Frontières 2024).

#### 2.1.5 Control Variables

In subsequent statistical modelling, we also controlled for the effects of respondents' gender (1 = man; 2 = woman), age, education level (levels of the International Standard Classification of Education), income (1 = lowest decile; 10 = highest decile within country), religiousness (0 = not at all religious; 10 = very religious), and ideological orientation (0 = left; 10 = right). On the country level, we applied social development as a control variable quantified by the Inequality‐Adjusted Human Development Index for 2020 (IHDI, United Nations 2024) to distinguish the state of media freedom from general social development.

### 2.2 Analysis and Results

Our assumptions were tested by multilevel structural equation modelling with the MPlus 8.5 software (Muthén and Muthén 1998–2017). For that, we applied full‐information Bayesian estimation because it results in more reliable parameter estimates compared to traditional frequentist methods in multilevel modelling (Hox et al. 2012). The estimation ran with two Markov chain Monte Carlo chains and 20,000 iterations, with the first half of those as the burn‐in phase. We used N(0, ∞) priors for regression coefficients and IG(0, ∞) priors for variances which work well in case of large samples (Asparouhov and Muthén 2010). As a first step, the variance of each individual‐level ESS variable was decomposed into a ‘within’ and a ‘between’ part, the former showing the part of the variance at the level of individuals (within countries), and the latter indicating the part that varies between countries. The estimated ‘within’ and ‘between’ variances of the variables are reported in Table 2 along with their descriptive statistics. As the second step, a multilevel moderated mediation model was set up. The within‐level was based exclusively on the ‘within’ variances of the ESS variables. Here, all three indicators of political support were predicted by perceived media freedom and authoritarianism, while the former was also regressed on the latter. These relationships were controlled for the effects of the control variables. The effect of authoritarianism on perceived media freedom was set as a random slope that could vary between countries. This random parameter was then regressed on the WPFI and IHDI indices on the ‘between’ level of the model to show their moderating effects.

**TABLE 2:** Descriptive statistics and estimated ‘within’ and ‘between’ variances.

Variable	Mean	Standard deviation	Estimated ‘within’ variance	SD	*p*	Estimated ‘between’ variance	SD	*p*
Authoritarianism—obedience and respect	3.464	1.158	1.235	0.007	*p* < 0.001	0.131	0.040	*p* < 0.001
Authoritarianism—loyalty	2.865	1.083	1.101	0.006	*p* < 0.001	0.080	0.025	*p* < 0.001
Authoritarianism—strong leader	2.505	3.127	8.502	0.050	*p* < 0.001	1.408	0.432	*p* < 0.001
Perceived media freedom	6.611	2.909	7.002	0.041	*p* < 0.001	1.506	0.461	*p* < 0.001
Political trust—parliament	4.498	2.730	6.296	0.037	*p* < 0.001	1.449	0.445	*p* < 0.001
Political trust—legal system	5.231	2.830	6.341	0.037	*p* < 0.001	2.002	0.611	*p* < 0.001
Political trust—police	6.236	2.619	5.875	0.034	*p* < 0.001	1.352	0.413	*p* < 0.001
Satisfaction—democracy	5.252	2.685	6.044	0.035	*p* < 0.001	1.498	0.448	*p* < 0.001
Satisfaction—government	4.357	2.669	6.362	0.037	*p* < 0.001	1.067	0.324	*p* < 0.001
Gender (*N*—woman)	31,285	—	0.248	0.001	*p* < 0.001	0.001	0.00	*p* < 0.001
Age	50.472	18.553	339.439	1.979	*p* < 0.001	7.388	2.330	*p* < 0.001
Education level	4.261	1.797	3.046	0.018	*p* < 0.001	0.260	0.080	*p* < 0.001
Household income	5.537	2.725	7.119	0.046	*p* < 0.001	0.340	0.105	*p* < 0.001
Religiousness	4.456	3.154	8.768	0.051	*p* < 0.001	1.278	0.391	*p* < 0.001
Left–right ideology	5.009	2.432	5.666	0.035	*p* < 0.001	0.248	0.076	*p* < 0.001
WPFI	79.957	8.377	—	—	—	—	—	—
IHDI	0.824	0.060	—	—	—	—	—	—

*Note*: Estimates are median points of the posterior Bayesian distribution.

Abbreviations: IHDI, inequality‐adjusted human development index; SD, standard deviations of the posterior median distribution; WPFI, World Press Freedom Index.

**FIGURE 1:**
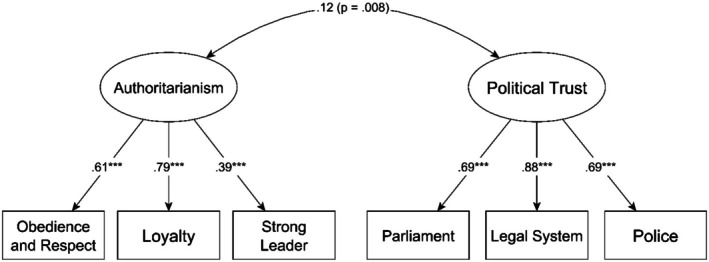
Measurement model for (within‐level) authoritarianism and political trust. The numbers on the arrows are standardised estimates; ***p < 0.001.

As both authoritarianism and political trust consisted of three indicator items, these were constructed as latent variables based only on the shared ‘within’ variances of the indicators. The measurement qualities of these latent variables were tested in a separate multilevel model with maximum likelihood estimation due to the absence of traditional fit indices for Bayesian multilevel models. At the ‘within’ level, two latent variables—authoritarianism and political trust—were created based on the ‘within’ variance parts of their indicator variables and allowed to correlate. Additionally, at the ‘between’ level, the ‘between’ variances of the same indicators were permitted to correlate to prevent artificially inflated model fit. This measurement model demonstrated adequate fit to the data, supporting the appropriate measurement qualities of the latent variables. (*χ* ^2^ = 248.23; df = 8; CFI = 0.969; RMSEA = 0.022; SRMR_within_ = 0.040; SRMR_between_ = 0.000; see also Figure 1).

**FIGURE 2:**
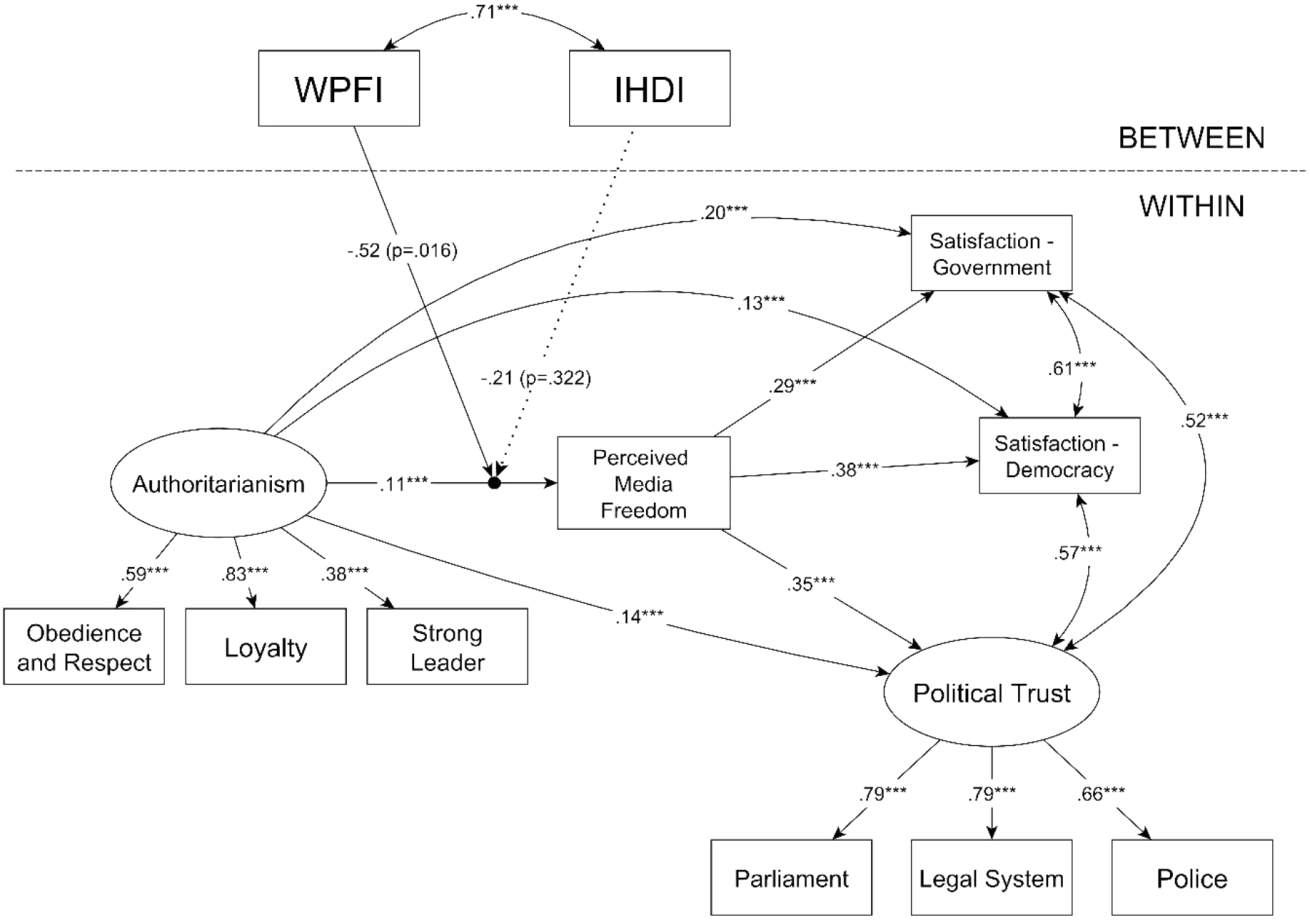
Multilevel moderated mediation model with cross‐level interactions. The numbers on the arrows are standardised estimates. The effects of the control variables are not displayed. IHDI, Inequality‐adjusted Human Development Index; WPFI, World Press Freedom Index. ***p < 0.001.

**FIGURE 3:**
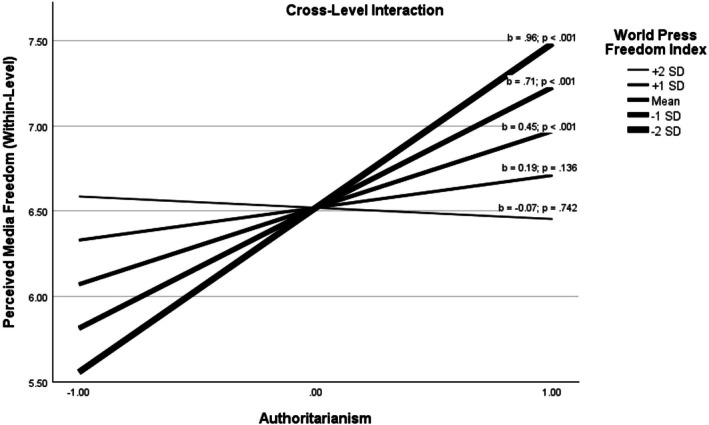
Simple slopes for the relationship between authoritarianism and (within‐level) perceived media freedom at different levels of the World Media Freedom Index.

The most important results of the final moderated mediation multilevel model are shown in Figure 2, and the detailed results are reported in Table 3. Our analysis showed that perceived media freedom had a significant positive statistical effect on each political support indicator (political trust: *b* = 0.26; *p* < 0.001; satisfaction with the government: *b* = 0.28; *p* < 0.001; satisfaction with democracy: *b* = 0.35; *p* < 0.001), and authoritarianism also showed a positive relationship with perceived media freedom (*b* = 0.45; *p* < 0.001). Based on these relationships, authoritarianism had a significant indirect statistical effect on each political support indicator through perceived media freedom (political trust: *b* = 0.12; *p* < 0.001; satisfaction with the government: *b* = 0.13; *p* < 0.001; satisfaction with democracy: *b* = 0.16; *p* < 0.001). Most importantly, the random effect of authoritarianism on perceived media freedom was significantly moderated by the WPFI (*b* = −0.03; *p* = 0.016) but not by the IHDI (*b* = −1.64; *p* = 0.322). To probe this moderation further, a series of simple slope analyses was conducted, which are reported in Table 4. These results show that the statistical effect of authoritarianism on perceived media freedom gradually increases with the drop of the WPFI and turns non‐significant at higher levels of the WPFI (see also Figure 3). The same could be observed in the case of the indirect associations between authoritarianism and the three indicators of political support.

**TABLE 3:** Results of multilevel model predicting political support.

Model parameter	Unstandardised estimate	SD	95% CI LB	95% CI UB	Standardised estimate	SD	95% CI LB	95% CI UB	*p*
Factor scores									
Authoritarianism—obedience and respect	1.00	—	—	—	0.591	0.005	0.582	0.600	< 0.001
Authoritarianism—loyalty	1.328	0.021	1.288	1.369	0.830	0.006	0.819	0.840	< 0.001
Authoritarianism—strong leader	1.689	0.023	1.645	1.735	0.382	0.005	0.372	0.391	< 0.001
Political trust—parliament	1.00	—	—	—	0.791	0.002	0.786	0.795	< 0.001
Political trust—legal system	0.997	0.006	0.984	1.009	0.785	0.002	0.780	0.790	< 0.001
Political trust—police	0.810	0.006	0.798	0.822	0.663	0.003	0.657	0.668	< 0.001
Correlations									
Political trust—satisfaction with democracy	2.239	0.023	2.194	2.285	0.570	0.004	0.563	0.577	< 0.001
Political trust—satisfaction with government	2.150	0.025	2.102	2.198	0.523	0.004	0.515	0.531	< 0.001
Satisfaction with democracy—satisfaction with government	3.097	0.025	3.047	3.147	0.607	0.003	0.602	0.612	< 0.001
WPFI—IHDI	0.441	0.172	0.220	0.889	0.708	0.103	0.454	0.856	< 0.001
Regression coefficients									
Authoritarianism—perceived media freedom	0.449	0.076	0.300	0.599	0.110	0.005	0.099	0.120	< 0.001
Authoritarianism—political trust	0.427	0.017	0.393	0.461	0.141	0.005	0.131	0.151	< 0.001
Authoritarianism—satisfaction with democracy	0.467	0.019	0.429	0.505	0.125	0.005	0.116	0.134	< 0.001
Authoritarianism—satisfaction with government	0.775	0.021	0.735	0.815	0.202	0.005	0.193	0.211	< 0.001
Perceived media freedom—gender	−0.205	0.022	−0.248	−0.162	−0.039	0.004	−0.047	−0.030	< 0.001
Perceived media freedom—age	0.013	0.001	0.011	0.014	0.089	0.004	0.081	0.097	< 0.001
Perceived media freedom—education level	0.061	0.007	0.048	0.074	0.040	0.004	0.032	0.049	< 0.001
Perceived media freedom—income	0.066	0.005	0.056	0.075	0.067	0.005	0.057	0.076	< 0.001
Perceived media freedom—religion	0.005	0.004	−0.003	0.012	0.005	0.004	−0.003	0.014	0.208
Perceived media freedom—ideology	0.040	0.005	0.030	0.050	0.036	0.004	0.028	0.045	< 0.001
Political trust—gender	−0.025	0.017	−0.059	0.009	−0.006	0.004	−0.015	0.002	0.164
Political trust—age	−0.003	0.000	−0.004	−0.002	−0.029	0.004	−0.038	−0.020	< 0.001
Political trust—education level	0.141	0.005	0.131	0.152	0.124	0.005	0.115	0.133	< 0.001
Political trust—income	0.074	0.004	0.067	0.082	0.100	0.005	0.090	0.111	< 0.001
Political trust—religion	0.062	0.003	0.056	0.067	0.092	0.005	0.083	0.100	< 0.001
Political trust—ideology	0.026	0.004	0.018	0.033	0.031	0.005	0.022	0.040	< 0.001
Political trust—perceived media freedom	0.263	0.003	0.256	0.269	0.348	0.004	0.340	0.356	< 0.001
Satisfaction with democracy—gender	0.001	0.019	−0.035	0.039	0.000	0.004	−0.007	0.008	0.906
Satisfaction with democracy—age	−0.002	0.001	−0.003	−0.001	−0.014	0.004	−0.022	−0.006	0.002
Satisfaction with democracy—education level	0.067	0.006	0.055	0.078	0.047	0.004	0.040	0.055	< 0.001
Satisfaction with democracy—income	0.074	0.004	0.066	0.082	0.081	0.004	0.072	0.089	< 0.001
Satisfaction with democracy—religion	0.048	0.003	0.041	0.054	0.058	0.004	0.050	0.065	< 0.001
Satisfaction with democracy—ideology	0.059	0.004	0.051	0.068	0.057	0.004	0.049	0.066	< 0.001
Satisfaction with democracy—perceived media freedom	0.352	0.004	0.345	0.359	0.378	0.004	0.371	0.384	< 0.001
Satisfaction with government—gender	0.070	0.020	0.032	0.109	0.014	0.004	0.007	0.022	< 0.001
Satisfaction with government—age	0.001	0.001	0.000	0.003	0.011	0.004	0.002	0.019	0.004
Satisfaction with government—education level	0.081	0.006	0.069	0.093	0.056	0.004	0.048	0.064	< 0.001
Satisfaction with government—income	0.052	0.004	0.043	0.060	0.055	0.005	0.046	0.064	< 0.001
Satisfaction with government—religion	0.050	0.003	0.044	0.057	0.059	0.004	0.051	0.067	< 0.001
Satisfaction with government—ideology	0.057	0.004	0.049	0.066	0.054	0.004	0.045	0.062	< 0.001
Satisfaction with government—perceived media freedom	0.280	0.004	0.273	0.287	0.292	0.004	0.285	0.300	< 0.001
Cross‐level interactions									
Authoritarianism—WPFI	−0.031	0.013	−0.056	−0.006	−0.518	0.194	−0.853	−0.101	0.016
Authoritarianism—IHDI	−1.640	1.690	−4.977	1.679	−0.210	0.207	−0.595	0.211	0.322
Random slope residuals									
Authoritarianism—perceived media freedom	0.153	0.053	0.088	0.291	0.510	0.138	0.271	0.807	< 0.001
R2									
Perceived media freedom	—	—	—	—	0.043	0.002	0.039	0.047	< 0.001
Political trust	—	—	—	—	0.195	0.004	0.188	0.202	< 0.001
Satisfaction with democracy	—	—	—	—	0.190	0.003	0.185	0.196	< 0.001
Satisfaction with government	—	—	—	—	0.157	0.003	0.151	0.163	< 0.001

*Note*: Estimates are median points of the posterior Bayesian distributions.

Abbreviations: 95% CI LB, lower bound of the 95% confidence interval; 95% CI UB, upper bound of the 95% confidence interval; IHDI, Inequality‐adjusted Human Development Index; SD, standard deviation of the posterior Bayesian distribution; WPFI, World Press Freedom Index.

**TABLE 4:** Simple slopes for the effects of authoritarianism at different levels of the World Press Freedom Index.

Pathway	Level of WPFI	Unstandardised estimate	SD	95% CI LB	95% CI UP	*p*
A—PMF	+2 SD	−0.066	0.224	−0.509	0.379	0.742
A—PMF	+1 SD	0.190	0.130	−0.064	0.449	0.136
A—PMF	Mean	0.449	0.076	0.300	0.599	< 0.001
A—PMF	−1 SD	0.707	0.130	0.450	0.961	< 0.001
A—PMF	−2 SD	0.964	0.224	0.527	1.402	< 0.001
A—PMF—SD	+2 SD	−0.023	0.079	−0.179	0.134	0.742
A—PMF—SD	+1 SD	0.067	0.046	−0.023	0.158	0.136
A—PMF—SD	Mean	0.158	0.027	0.105	0.211	< 0.001
A—PMF—SD	−1 SD	0.249	0.046	0.158	0.338	< 0.001
A—PMF—SD	−2 SD	0.340	0.079	0.185	0.494	< 0.001
A—PMF—PT	+2 SD	−0.017	0.059	−0.133	0.100	0.742
A—PMF—PT	+1 SD	0.050	0.034	−0.017	0.118	0.136
A—PMF—PT	Mean	0.118	0.02	0.079	0.157	< 0.001
A—PMF—PT	−1 SD	0.185	0.034	0.118	0.252	< 0.001
A—PMF—PT	−2 SD	0.253	0.059	0.138	0.368	< 0.001
A—PMF—SG	+2 SD	−0.019	0.063	−0.143	0.106	0.742
A—PMF—SG	+1 SD	0.053	0.036	−0.018	0.126	0.136
A—PMF—SG	Mean	0.126	0.021	0.084	0.168	< 0.001
A—PMF—SG	−1 SD	0.198	0.036	0.126	0.269	< 0.001
A—PMF—SG	−2 SD	0.270	0.063	0.147	0.392	< 0.001

*Note*: Estimates are median points of the posterior Bayesian distributions.

Abbreviations: 95% CI LB, lower bound of the 95% confidence interval; 95% CI UB, upper bound of the 95% confidence interval; A, authoritarianism; PMF, perceived media freedom; PT, political trust; SD, satisfaction with democracy; SD, standard deviation of the posterior Bayesian distribution; SG, satisfaction with government.

## 3 Discussion

Our results show that authoritarianism is related to various dimensions of political support, partly through positive perceptions of media freedom. This mediating role is particularly pronounced in countries with lower actual media freedom levels. Essentially, authoritarianism is associated with a divergence between motivated subjective perceptions and reality, possibly leading authoritarians to ignore unpleasant truths about free speech. These findings align with previous research showing that motivational factors favouring the status quo can establish biased positive perceptions of established political systems (Hadarics and Kende 2025, Hadarics and Krekó 2025). But taking a step further, our results also indicate that in the case of the democratic principle of free speech, this ignorance helps authoritarians maintain their support for a political system that suits their preference for social unity without dissident voices.

Authoritarianism has been known as one of the key dispositional traits serving as a psychological foundation for anti‐democratic and illiberal preferences (Duckitt 2022; Feldman et al. 2021). At the same time, even illiberal regimes are trying to maintain the illusion of democracy (Bozóki 2013; Roth 2009), which makes it more difficult for authoritarians to express openly restrictive policy suggestions about the public discourse. This difficulty can be solved by motivated ignorance or direct denial of any central limitations of media freedom or free speech, which, in turn, leads to the approval of the regime that frames itself as democratic but does not act so. This interpretation is in line with the literature on political support and trust, which shows that perceived democratic quality is an important base for political support (van der Meer 2018), but our results also suggest that such perceptions can be vulnerable to motivated biases stemming from dispositional characteristics like authoritarianism.

Future research should also take into account the content of public discourse. For instance, authoritarians may support hate speech prohibition even when the targets of hate speech are disliked by them, as openly expressing hateful statements goes against social conventions (Bilewicz et al. 2017). Nonetheless, if authoritarians tend to establish biased perceptions about the public discourse, as our results suggest, they might underestimate the prevalence of hate speech in their endeavour to justify an illiberal status quo.

Although our results were based on representative data from 31 European nations and almost 60,000 respondents, they still represent a relatively small geographic area, Europe, where average media freedom is significantly wider compared to other regions worldwide. Future research should include more authoritarian regimes because, in certain cases, even the illusion of democracy could be unnecessary to preserve political power, which would make motivated misperception an ineffective strategy for justifying the political status quo. Finally, it is important to note that our study was based on a cross‐sectional international survey database and, as such, is not suitable for identifying causal effects between variables—only correlational relationships. Nevertheless, our study is not unique in this regard, as authoritarianism is typically considered, both theoretically and empirically, to be a foundation for more specific beliefs and attitudes. This directional effect is also supported by longitudinal findings (for a review, see Duckitt 2022; Feldman and Weber 2023).

## 4 Conclusion

Dispositional authoritarianism has been widely associated with preferences that align with anti‐democratic and illiberal values, including support for restrictions on free speech and centralised media control. The present findings suggest that authoritarianism is also linked to more favourable perceptions of media freedom, particularly in contexts where such freedom is limited. This pattern may reflect a broader tendency among authoritarians to perceive political systems in ways that align with their preference for social cohesion and order. These perceptions appear to coincide with higher levels of political support, even in environments where democratic principles are under strain. This indicates that authoritarianism can contribute to democratic backsliding and damage open and free political communication not only by establishing anti‐democratic policy preferences but also by initiating a motivated misperception and ignorance of problems with the actual functioning of democratic principles like freedom of speech.

## Author Contributions

**Márton Hadarics:** conceptualization, research design planning, data analysis and manuscript preparation were performed by the author.

## Ethics Statement

The author has nothing to report.

## Conflicts of Interest

The author declares no conflicts of interest.

## Data Availability

The author confirm that the data supporting the findings of this study is available within the reference list of the article.
